# Serum Fetuin-B Levels Are Elevated in Women with Metabolic Syndrome and Associated with Increased Oxidative Stress

**DOI:** 10.1155/2021/6657658

**Published:** 2021-10-04

**Authors:** Shiyao Xue, Hongdong Han, Shunli Rui, Mengliu Yang, Yizhou Huang, Bin Zhan, Shan Geng, Hua Liu, Chen Chen, Gangyi Yang, Ling Li

**Affiliations:** ^1^Key Laboratory of Diagnostic Medicine (Ministry of Education) and Department of Clinical Biochemistry, College of Laboratory Medicine, Chongqing Medical University, 400016 Chongqing, China; ^2^Department of Endocrinology, The Second Affiliated Hospital, Chongqing Medical University, Chongqing 400010, China; ^3^Department of Endocrinology, Multidisciplinary Diabetic Foot Medical Center, Chongqing Emergency Medical Center, Chongqing University Central Hospital, Chongqing University, Chongqing, China; ^4^Endocrinology, SBMS, Faculty of Medicine, University of Queensland, Brisbane 4072, Australia; ^5^The Thirteenth People's Hospital of Chongqing, Chongqing 400016, China; ^6^Department of Pediatrics, University of Mississippi Medical Center, 2500 North State Street, Jackson, Mississippi, MS 39216-4505, USA

## Abstract

Previous studies on serum fetuin-B (fetuin-like protein IRL685) have investigated its association with T2DM; however, the reason for the variation in serum fetuin-B and its regulatory factors in metabolic disease remain unclear. Here, we evaluated serum fetuin-B levels in women with newly diagnosed MetS and performed multiple interventions to investigate the role of fetuin-B in the pathogenesis of MetS. Serum fetuin-B levels were assessed using ELISA. Bioinformatics analysis was performed to analyze fetuin-B-related genes and signaling pathways. Additionally, oxidative stress parameters were measured in the *in vitro* study. For subgroup analyses, we performed EHC, OGTT, and treatment with a GLP-1RA to investigate the regulatory factors of serum fetuin-B. We found that in comparison with healthy subjects, serum fetuin-B levels were markedly increased in women with MetS. Further, serum fetuin-B showed a positive correlation with WHR, FAT%, TG, FBG, HbA1c, FIns, HOMA-IR, VAI, and LAP. Bioinformatics analysis revealed that most fetuin-B-related core genes were involved in cholesterol metabolism and fat decomposition. Consistent with this finding, multivariate regression analysis showed that triglyceride content and WHR were independently associated with serum fetuin-B. We also observed that serum fetuin-B levels were markedly elevated in healthy subjects after glucose loading and in women with MetS during EHC. *In vitro*, overexpression of fetuin-B promoted oxidative stress in HepG2 cell. After 6 months of treatment with a GLP-1RA, serum fetuin-B levels in women with MetS decreased following an improvement in metabolism and insulin sensitivity. Therefore, serum fetuin-B is associated with MetS, which may serve as a biomarker of oxidative stress. This trial is registered with ChiCTR-OCC-11001422.

## 1. Introduction

Metabolic syndrome (MetS) is characterized by several symptoms, including insulin resistance (IR), abdominal obesity, hypertension, and dyslipidemia [1]. Over the past few decades, MetS and obesity have developed into global epidemics, attributable to high-fat diet and sedentary lifestyle, placing an enormous economic burden on healthcare systems [2]. The risk of cardiovascular disease and type 2 diabetes mellitus (T2DM) has been reported to increase by 2- and 5-fold in patients with MetS, respectively [3, 4]. Furthermore, MetS increases all-cause mortality from 1.30- to 1.70-fold, with women showing a higher incidence than men [5]. Therefore, the pathogenesis of MetS and how its components interact remain uncertain, and there is a lack of consistent treatment recommendation. For the optimal management of patients with MetS, it is thus critical to identify biomarkers that can accurately predict outcomes and therapeutic responses.

Fetuin-like protein IRL685 (fetuin-B) was first identified as the second member of the cystatin superfamily of cysteine protease inhibitors in 2000. It shows 22% homology with fetuin-A [6]. Fetuin-B is encoded by the FETUB gene, which has a chromosomal localization of 3q27.3 with eight exons, and mutations in this region have previously been confirmed to be prone to MetS and diabetes [6]. Further, fetuin-B expression levels have been reported to be higher in hepatocytes from mice with liver steatosis, impairing insulin action in myotubes and hepatocytes. In addition, fetuin-B silencing improved glucose tolerance in obese mice but did not affect their body weight, which suggests that fetuin-B regulates blood glucose levels via an insulin-independent mechanism, possibly through glucose effectiveness [7, 8]. In clinical studies, serum fetuin-B was found to be strongly associated with triglyceride (TG) content in the liver and early insulin secretion stimulated by glucose [9, 10]. It has also been reported that in comparison with healthy controls, fetuin-B levels are substantially increased in patients with nonalcoholic fatty liver disease, T2DM, polycystic ovary syndrome, and gestational diabetes mellitus [11–13]. Therefore, previous studies have suggested that fetuin-B, as an adipokine or hepatokine, is closely related to glucose and lipid metabolism. However, the association between fetuin-B and MetS and its mechanism remain unclear.

In this study, we measured serum fetuin-B levels in healthy individuals and women newly diagnosed with MetS. A preprint for this part of the study has been published [14]. To investigate the association between serum fetuin-B and MetS as well as IR, we performed multiple intervention experiments, including euglycemic-hyperinsulinemic clamps (EHCs), oral glucose tolerance tests (OGTTs), and treatment with a glucagon-like peptide-1 receptor agonist (GLP-1RA). Furthermore, we investigated the effect of fetuin-B on FFA-induced oxidative stress *in vitro*.

## 2. Materials and Methods

### 2.1. Participants and Inclusion/Exclusion Criteria

Overall, 377 Chinese women (192 with MetS, 185 healthy subjects; age, 38 ± 15 years; body mass index (BMI), 23.7 ± 4.2 kg/m^2^) were recruited from outpatients attending the Department of Endocrinology at the Second Affiliated Hospital, Chongqing Medical University, as well as from the community or universities via advertisements or routine medical examinations performed between December 2016 and December 2018. The diagnostic criteria of MetS were based on the United States National Cholesterol Education Program Expert Panel Adult Treatment Panel III criteria [15]. Participants were diagnosed with MetS if they showed ≥3 of the following: (1) central obesity (waist circumference (WC): Asian women, ≥80 cm), (2) hypertension (≥130/85 mmHg), (3) hyperglycemia (fasting glucose ≥ 5.6 mmol/L or T2DM), (4) elevated plasma TG levels (≥1.69 mmol/L), and (5) low levels of high-density lipoprotein-cholesterol (HDL-C: women, <1.29 mmol/L). The participants were classified as those having normal glucose tolerance, impaired glucose tolerance, or T2DM, using the diagnostic criteria of the American Diabetes Association [16]. The exclusion criteria included heart, hepatic, or renal failure; presence of malignant tumors, type 1 diabetes, acute infection, or other chronic metabolic diseases; pregnancy; and long-term use of steroids. In this study, all participants with MetS were newly diagnosed, and there was no involvement of any drug treatment, physical exercise, or diet control. The healthy subjects had no history of other diseases, hypertension, or family history of diabetes, and their blood glucose levels were normal. All study protocols were approved by the Human Research Ethics Committee of Chongqing Medical University, and all experiments were performed in accordance with the Declaration of Helsinki. Written informed consent was obtained from all participants.

### 2.2. Anthropometric Examinations and Biochemical Measurements

After an overnight fast for at least 12 h, anthropometric measurements were made and blood samples were obtained by professionals from all participants. Body measurements (weight, height, WC, hip circumference (HC), blood pressure, and fat percentage *in vivo* (FAT%)) and biochemical indices, including levels of fasting blood glucose (FBG), 2 h post-OGTT glucose (2 h-BG), insulin, glycosylated hemoglobin (HbA1c), TG, total cholesterol (TC), HDL-C, low-density lipoprotein cholesterol (LDL-C), and free fatty acids (FFA), were measured, as previously reported [17]. We also subjected all women to a 75 g, 2 h OGTT, according to the American Diabetes Association criteria [16].

### 2.3. Measurement of Serum Fetuin-B Levels

Serum fetuin-B levels were determined using a commercial enzyme-linked immunosorbent assay kit (RayBiotech, Inc., Norcross, GA, USA), as the manufacturer's instructions. The detection limit of this kit was 4.0 ng/mL; the intra- and interassay coefficient of variation was 10% and 12%, respectively.

### 2.4. Bioinformatic Analysis

#### 2.4.1. Protein-Protein Interaction (PPI) Network Construction and REACTOME Analysis

The Search Tool (v11.0) for the Retrieval of Interacting Genes (STRING) database was used to construct the PPI network [18]. An interaction score of 0.4 was used as the cut-off standard, and the PPI network was visualized. The STRING database was used for REACTOME enrichment analysis to screen out signal pathways and genes related to metabolism [19]. The false discovery rate (FDR) < 0.01 indicated statistical significance in REACTOME analysis.

#### 2.4.2. Gene Ontology (GO) and Kyoto Encyclopedia of Genes and Genomes (KEGG) Analysis

We used the clusterProfiler package to perform GO and KEGG pathway analyses [20]. A list of GO and KEGG annotation terms was thus obtained. Further, we categorized all genes to the biological processes (BP), cellular components (CC), and molecular functions (MF) of GO categories. *p* < 0.05 indicated statistical significance in the case of GO and KEGG terms.

### 2.5. In Vitro Study

#### 2.5.1. Cell Culture and Treatment

HepG2 cells were cultured in DMEM supplemented by 10% fetal bovine serum as previously reported [21]. The cells were transfected with plasmid expressing fetuin-B (pcDNA3.1-fetuin-B, GenePharma, Inc. Shanghai, China) or control plasmid (pcDNA3.1) for 24 h. To induce oxidative stress, HepG2 cells were exposed to 1 mM fatty acid mixtures (FFAs, oleic acid: palmitate, 2 : 1) for another 24 h as previously reported [21].

#### 2.5.2. Determination of Oxidative Stress Parameters

The activities of antioxidant enzymes including superoxide dismutase (SOD), glutathione (GSH), and malondialdehyde (MDA) were measured by their specific assay kits (Beyotime, Inc., Shanghai, China), according to the manufacturer's instructions. To investigate intracellular reactive oxygen species (ROS) formation, FFA-treated HepG2 cells were incubated with 10 *μ*M dichloro-dihydro-fluorescein diacetate (DCFH-DA) at 37°C for 20 min. Fluorescence of ROS in the cells was recorded with a fluorescence microscope (Olympus Corporation, Tokyo, Japan), and intensity was analyzed using ImageJ software.

### 2.6. EHC

For subgroup analysis, EHC was performed in 16 women with MetS and 27 healthy subjects as previously reported [17]. A venous channel was established in the antecubital vein to infuse insulin and glucose, and another catheter was implanted into the dorsal vein of the contralateral hand for blood sampling. Regular human insulin (1 mU/kg/min) was infused for 2 h, and 20% glucose was infused to maintain blood glucose levels at the primary level. The blood glucose level was measured every 15 min during EHC to guide the glucose infusion rate. During the steady state of the clamp, the glucose infusion rate was equal to the glucose disposal rate and related to body weight (*M* value). Blood samples were collected at 0, 80, 100, and 120 min to analyze serum fetuin-B levels and other parameters. The blood samples were centrifuged to separate serum and stored at −80°C until analysis.

### 2.7. Intervention Therapy with Liraglutide (GLP-1RA)

Twenty-four obese women with MetS were recruited from outpatients attending the Department of Obesity at the Second Affiliated Hospital, Chongqing Medical University. The inclusion criteria were age 18–35 years, BMI of 25–35 kg/m^2^, and meeting the diagnostic criteria of MetS which have been described previously [15]. The exclusion criteria included the presence of medullary thyroid carcinoma or severe gastrointestinal diseases, family history of thyroid tumor, pregnancy, and recent history of medication. These individuals have voluntarily participated in the GLP-1RA intervention study for 6 months. The initial dose of liraglutide taken before breakfast was 0.6 mg/d; the dose was increased by 0.6 mg/d every week until it reached 1.8 mg/d. All participants provided informed consent and were aware of the potential side effects of liraglutide before treatment. Anthropometric measurements, biochemical examinations, OGTTs, and EHCs were performed as described above before treatment and also at weeks 12 and 24.

### 2.8. Calculations

BMI (kg/m^2^) was calculated as weight (kg) divided by height squared (m^2^). Waist-to-hip ratio (WHR) was calculated as follows: WC (cm)/HC (cm). The *M* value was calculated as glucose infusion rate divided by body weight, as previously described [17]. Homeostasis model assessment of insulin resistance (HOMA-IR) was calculated as follows: [fasting insulin (FIns, mU/L) × FBG (mmol/L)]/22.5. The cut-off value of IR was HOMA-IR > 3 [22]. Visceral adiposity index (VAI) was calculated as [WC (cm)/(36.58 + BMI (kg/m^2^) × 1.89)] × [TG (mmol/L)/0.81] × [1.52/HDL‐C (mmol/L)]. Finally, lipid accumulation product (LAP) in women was calculated as follows: [WC (cm) − 58] × TG (mmol/L) [23, 24].

### 2.9. Statistical Analysis

SPSS v24.0 (SPSS, Chicago, IL) was used for statistical analyses. Values are expressed as the mean ± SD or SME, or median with interquartile range. Before analysis, logarithmic conversion was conducted for variables with nonnormal distribution. Student's *t*-test, nonparametric tests, or analysis of variance was used to compare differences between groups. Simple and partial correlation analyses were conducted to explore the relationships between variables and serum fetuin-B. The variables that showed an independent association with serum fetuin-B levels were evaluated using multiple linear regression. Binary logistic regression analysis was used to examine the association between serum fetuin-B and MetS. The change in fetuin-B levels in women with MetS was analyzed using the row mean score and Cochran–Armitage trend test. When data were compared with the control group, *p* < 0.05 indicated a significant difference.

## 3. Results

### 3.1. Anthropometric and Biochemical Parameters and Serum Fetuin-B Levels

**Figure 1:**
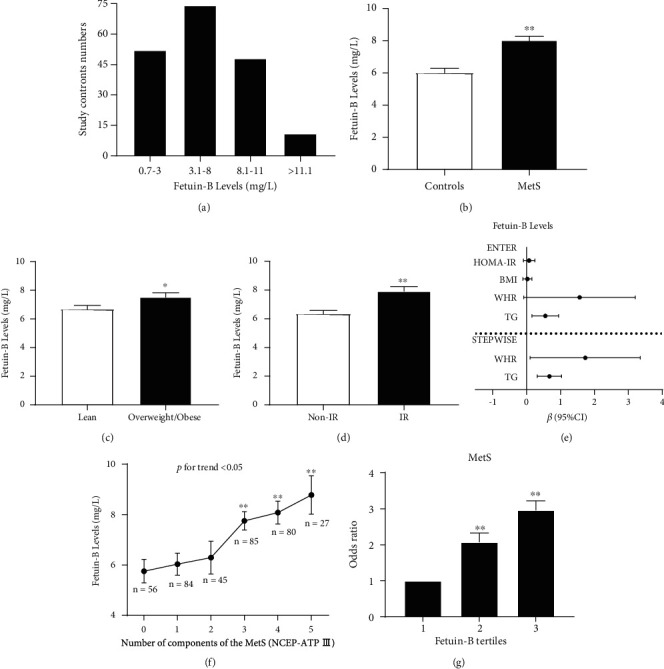
Serum fetuin-B levels in the study population. (a) Distribution of serum fetuin-B in 185 healthy women. (b) Serum fetuin-B levels in MetS and healthy subjects. (c) Serum fetuin-B levels according to BMI (lean: BMI < 24 kg/m^2^; overweight/obese: BMI ≥ 24 kg/m^2^). (d) Serum fetuin-B levels, according to HOMA-IR (IR: HOMA‐IR > 3; non-IR: HOMA‐IR ≤ 3). (e) All factors and stepwise multiple regression analyses of the serum fetuin-B and MetS in study individuals. (f) Serum fetuin-B levels in relation to the number of MetS components. (g) The odds ratio of having MetS in different tertiles of serum fetuin-B (tertile 1, ≤5.49 mg/L; tertile 2, 5.49-8.58 mg/L; tertile 3, >8.58 mg/L). Data were means ± SME. ^∗^*p* < 0.05 or ^∗∗^*p* < 0.01 vs. controls, lean, no IR, or tertile 1.

In this cross-sectional study, the distribution range of serum fetuin-B levels was 1.08–10.30 mg/L for most healthy subjects (83.2%, Figure 1(a)).  Table 1 shows the main clinical features and metabolic parameters of all participants (average age, 37.9 ± 15.6 years). In comparison with healthy subjects (6.01 ± 3.94 mg/L), women with MetS showed a significant increase in serum fetuin-B levels (8.03 ± 3.75 mg/L, *p* < 0.001, Table 1, Figure 1(b)). After adjustment for age and BMI, this increase remained significant. In comparison with healthy subjects, age, BMI, FAT%, WHR, blood pressure, blood lipids (including TG, TC, LDL-C, and FFA), FBG, 2 h-BG, FIns, 2 h insulin after glucose overload (2 h-Ins), HbA1c, HOMA-IR, VAI, and LAP were significantly higher in women with MetS, while HDL-C was lower (*p* < 0.001 for all, Table 1). In addition, there was a statistically significant increase in serum fetuin-B levels in overweight/obese women (*n* = 161, BMI ≥ 24 kg/m^2^) than in lean women (*n* = 216, BMI < 24 kg/m^2^; 7.52 ± 4.01 vs. 6.68 ± 3.91 mg/L, *p* < 0.05; Figure 1(c)). To assess the association between serum fetuin-B levels and IR, we classified all subjects into IR (HOMA-IR > 3) and no IR (HOMA-IR ≤ 3) groups. In comparison with the no IR group (6.33 ± 3.95 mg/L), serum fetuin-B levels were markedly elevated in the IR group (7.92 ± 4.04 mg/L, *p* < 0.01; Figure 1(d)).

### 3.2. Association between Fetuin-B and Other Variables

Linear correlation analysis showed that there was a significant positive correlation between fetuin-B and obesity- and lipid-related (BMI, WHR, FAT%, TG, LAP, and VAI) as well as glucose-related (HbA1c, FBG, 2 h-BG, FIns, 2 h-Ins, and HOMA-IR) (*p* < 0.05 or *p* < 0.01) parameters in all subjects, but no correlation was observed between fetuin-B and HDL-C or FFA (Table S1). Moreover, TG and WHR were independently associated with serum fetuin-B, as evident via multiple regression analysis (Figure 1(e), Table S1). The multiple regression equation was *Y*_fetuin‐B_ = 4.381 + 0.674*X*_TG_ + 1.731*X*_WHR_.

### 3.3. Relationship between Serum Fetuin-B and MetS

Logistic regression analysis demonstrated that serum fetuin-B was related to MetS (odds ratio OR, 1.150; 95% confidence interval (CI), 1.086–1.217; *p* < 0.01). This relationship persisted even after age, BMI, FAT%, HbA1c, FIns, TC, LDL, and FFA, and other possible confounding factors were controlled (Table S2). Further, the row mean score and Cochran–Armitage trend test showed a significant linear trend and independent correlation between serum fetuin-B levels and MetS (Table S3). Moreover, according to MetS components, we divided the mean levels of serum fetuin-B into six grades.  Figure 1(f) shows that with an increase in MetS components, serum fetuin-B levels progressively increased (*p* for trend <0.05). Individuals with 0, 1, 2, 3, 4, and 5 MetS components showed increasing serum fetuin-B levels (5.75 ± 3.49, 6.03 ± 4.00, 6.29 ± 4.38, 7.75 ± 3.39, 8.08 ± 4.05, and 8.78 ± 3.97 mg/L, respectively). Furthermore, we divided serum fetuin-B levels into tertile 1 (≤5.49 mg/L), 2 (5.49–8.58 mg/L), and 3 (>8.58 mg/L). The OR was calculated as an estimate of developing MetS. The risk of developing MetS in tertiles 2 and 3 was higher than that in tertile 1 (OR, 2.07; 95% CI, 1.25–3.43 for tertile 2; OR, 2.96; 95% CI, 1.78–4.94 for tertile 3; vs. tertile 1, *p* < 0.01 for all, Figure 1(g)).

### 3.4. Bioinformatic Analysis of Fetuin-B-Related Genes and Signaling Pathways

#### 3.4.1. PPI Network Construction

**Figure 2:**
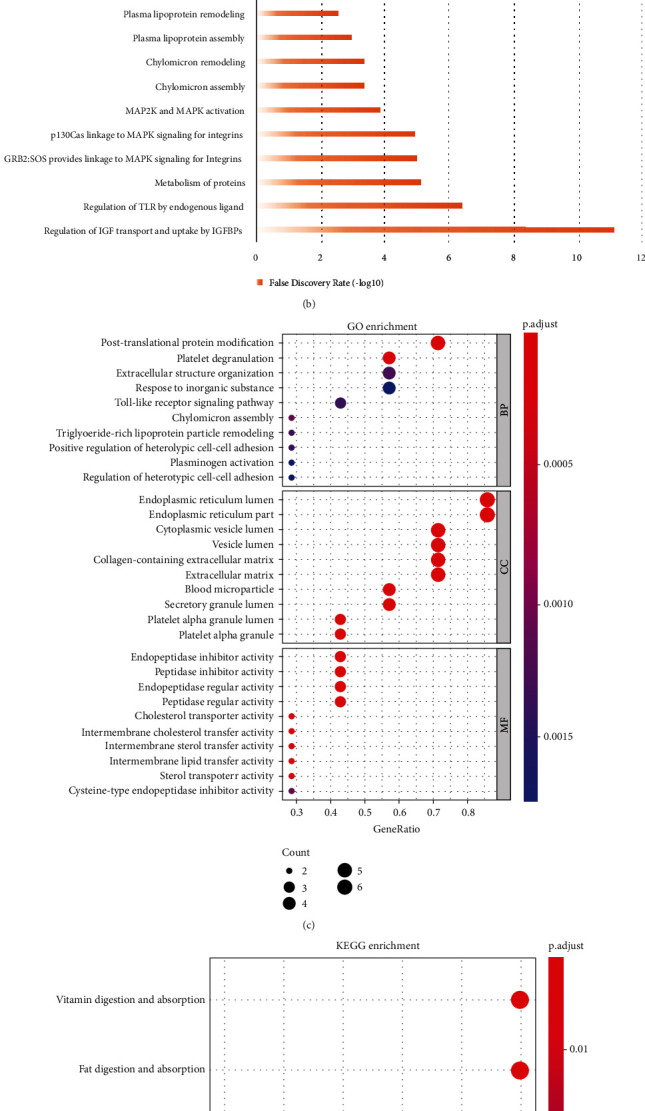
Bioinformatics analysis related to fetuin-B. (a) The PPI network through the keyword FETUB related to metabolism. (b) The enriched pathways of the REACTOME. The *X*-axis represents FDR. The *Y*-axis represents the pathway terms. The longer the bar means the more reliable the pathways. (c, d) The results of GO and KEGG analysis. The *X*-axis represents the ratio of involved genes, and the *Y*-axis represents GO and KEGG terms. The size of the bubbles indicates the number of genes involved, and each bubble represents a term. The darker the color, the smaller the *p* value.

We ranked the degree of genes enriched in metabolic pathways from high to low. The top six genes (fibrinogen alpha chain (FGA), apolipoprotein B-100 (APOB), apolipoprotein A-IV (APOA4), fibrinogen gamma chain (FGG), alpha-2-HS-glycoprotein (AHSG), and secreted phosphoprotein 24 (SPP2)) were used as the core genes for another round of enrichment (Figure 2(a)). Metabolic pathways were screened *via* REACTOME enrichment analysis, and the core genes enriched in those pathways were ranked (Figure 2(b)).

#### 3.4.2. GO Analysis

We used *p* < 0.05 as the screening condition and arranged the results from large degree to small degree. In the case of biological processes, proteins were mainly involved in posttranslational protein modification, toll-like receptor signaling pathway, chylomicron assembly, and TG-rich lipoprotein particle remodeling. In the case of cellular components, proteins were mainly enriched in the endoplasmic reticulum lumen, endoplasmic reticulum part, and cytoplasmic vesicle lumen. Finally, in the case of molecular function, proteins were mainly involved in endopeptidase inhibitor and regulator activity, cholesterol transporter activity, and intermembrane cholesterol transfer activity (Figure 2(c)).

#### 3.4.3. KEGG Pathway Analysis

We used *p* < 0.05 as the screening condition and ranked the *p* values from large to small. We found that proteins were predominantly enriched in pathways associated with vitamin digestion and absorption, fat digestion and absorption, cholesterol metabolism, complement and coagulation cascades, platelet activation, and staphylococcus aureus infection (Figure 2(d)).

### 3.5. Overexpression of Fetuin-B Aggravated Oxidative Stress in HepG2 Cells

It has been reported that metabolic disorder is related to oxidative stress [25]. Therefore, we investigated the effect of fetuin-B on FFA-induced oxidative stress in HepG2 cells.

**Figure 3:**
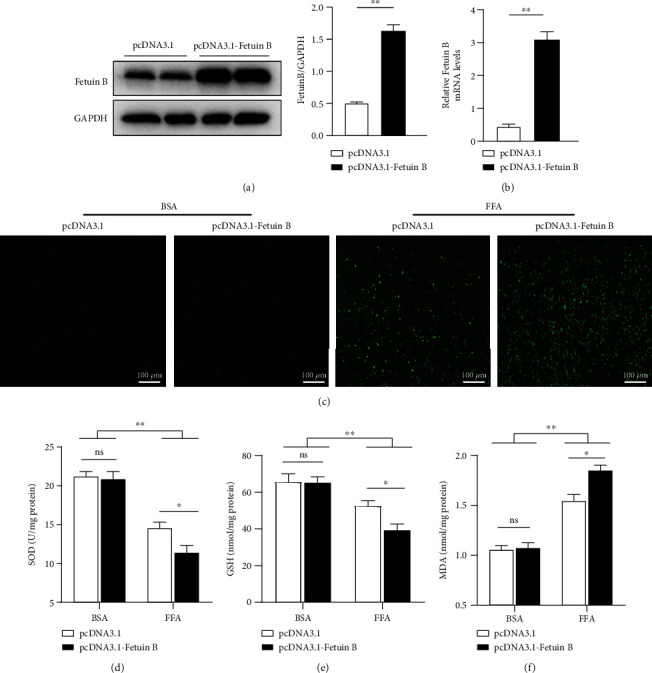
Fetuin-B exacerbated FFA-induced oxidative stress in HepG2 cells. HepG2 cells were transfected with pcDNA3.1-fetuin-B or pcDNA3.1 for 24 h and treated as indicated in the methods. (a) Fetuin-B protein expression. (b) Fetuin-B mRNA expression. (c) The level of intracellular ROS production. (d) The SOD activity. (e) The GSH production. (f) The MDA production. ROS: reactive oxygen species; SOD: superoxide dismutase; GSH: glutathione; MDA: malondialdehyde. The results were presented as the mean ± SD. ^∗^*p* < 0.05 and ^∗∗^*p* < 0.01.

As expected, the expression of fetuin-B protein and mRNA was significantly increased in HepG2 cells transfected with pcDNA3.1-fetuin-B (Figures 3(a) and 3(b)). As shown in Figure 2(c), overexpression of fetuin-B in HepG2 cells significantly increased intracellular ROS production induced by FFAs, compared with that in control cells. Furthermore, in FFA-treated HepG2 cells, overexpression of fetuin-B significantly decreased SOD and GSH levels but increased MDA levels (Figures 3(d)–3(f)). These results indicated that fetuin-B increases oxidative stress in HepG2 cells.

### 3.6. Effects of Hyperglycemia and Hyperinsulinemia on Serum Fetuin-B

**Figure 4:**
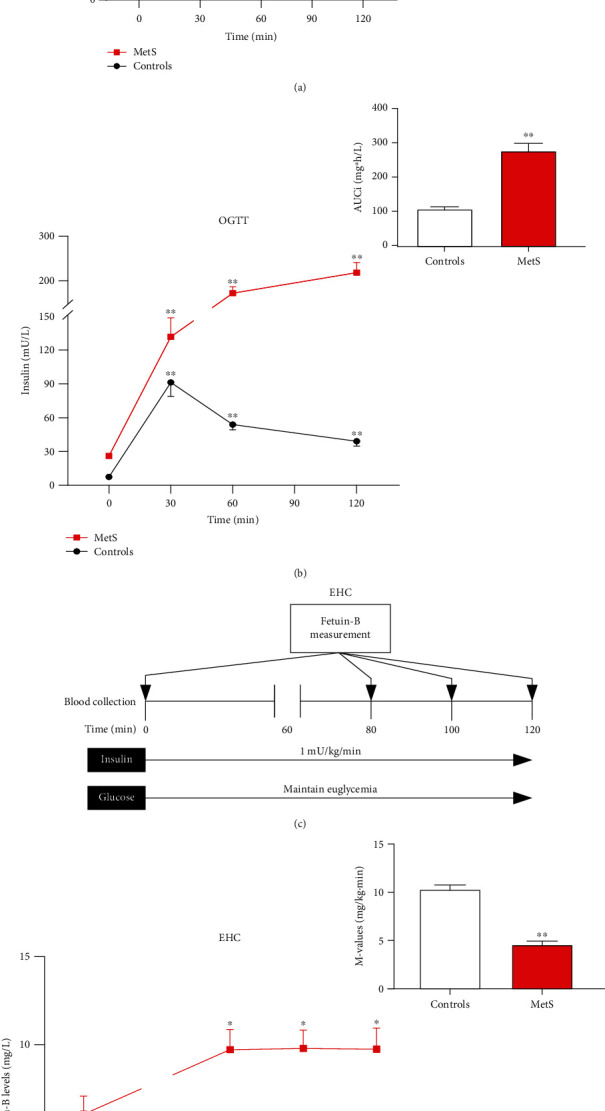
Serum fetuin-B levels in interventional studies. (a) Time course of changes in serum fetuin-B levels in healthy (*n* = 15) and MetS subjects (*n* = 28) during the OGTT and the area under the curve for serum fetuin-B (AUC_f_). (b) Glucose-stimulated insulin secretion curve in healthy (*n* = 15) and MetS (*n* = 28) subjects during the OGTT and the area under the curve for insulin (AUC_i_). (c) EHC protocol. (d) Time course of serum fetuin-B changes and the *M* values in healthy (*n* = 16) and MetS subjects (*n* = 27) during the EHC. Data were means ± SME. ^∗^*p* < 0.05 or ^∗∗^*p* < 0.01 vs. control or baseline.

To understand the effects of glucose load on the circulating levels of fetuin-B, we performed OGTTs. In healthy women, serum fetuin-B levels were significantly higher after glucose challenge compared with the basal value, peaking at 30 min (from 3.34 ± 2.96 to 10.15 ± 5.17 mg/L) and remaining stable until the end of the experiment (Figure 4(a)). However, in patients with MetS, glucose load did not cause any significant changes in serum fetuin-B levels (Figure 4(a)). In comparison with healthy subjects, the area under the curve for fetuin-B (AUC_f_) was significantly increased in women with MetS (Figure 4(a)). During OGTTs, glucose-stimulated insulin secretion curves showed that the level of insulin secretion in women with MetS was significantly higher than that in healthy subjects and showed a peak delay; moreover, the area under the curve for insulin (AUC_i_) was increased in comparison with healthy subjects, which also confirmed the existence of IR in patients with MetS (Figure 4(b)). We then performed EHCs to further explore the factors affecting the secretion of serum fetuin-B (Figure 4(c)). In response to hyperinsulinemia, serum fetuin-B levels significantly increased in patients with MetS, whereas there was no change in healthy subjects (Figure 4(d)). Meanwhile, women with MetS showed lower *M* values than healthy women (4.47 ± 1.88 vs. 10.23 ± 2.79 mg/kg/min, *p* < 0.01; Figure 4(d)). These results indicated that patients with MetS showed IR and that serum fetuin-B secretion might be regulated by circulating insulin levels *in vivo*.

### 3.7. Effects of GLP-1RA Intervention on Serum Fetuin-B Levels

**Figure 5:**
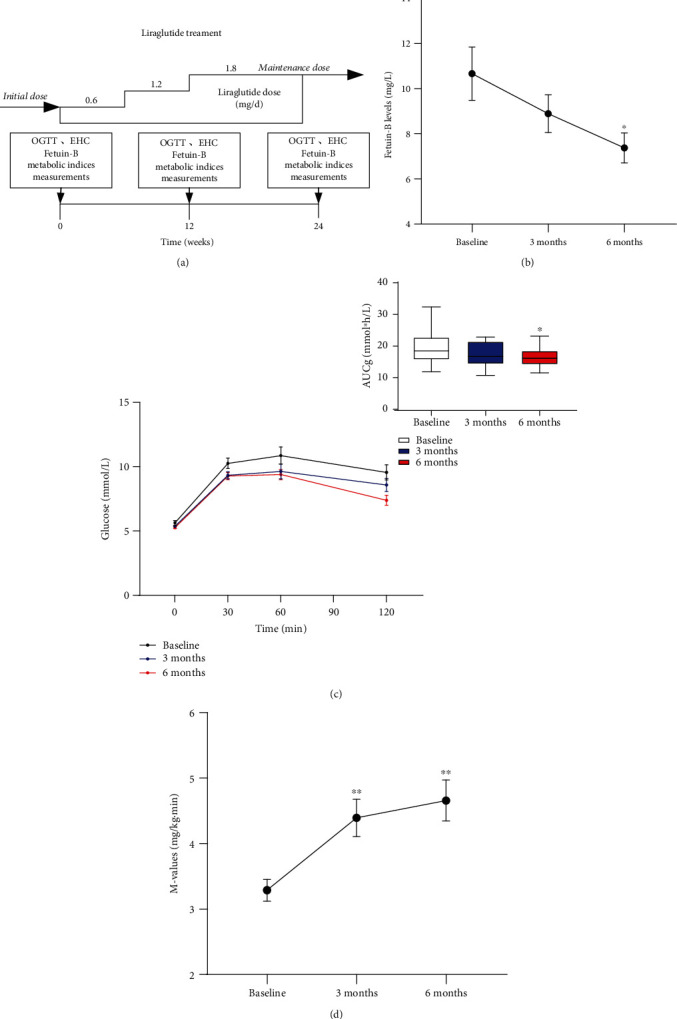
Effects of GLP-1RA treatment on serum fetuin-B and insulin sensitivity in MetS women. (a) Liraglutide treatment protocol. (b) Serum fetuin-B levels in MetS subjects after GLP-1RA treatment. (c) Blood glucose levels and the area under the curve for blood glucose (AUCg) in MetS subjects during the OGTT after GLP-1RA treatment. (d) Changes of *M* value in MetS subjects during the EHC after GLP-1RA treatment. Data were means ± SME. ^∗^*p* < 0.05 or ^∗∗^*p* < 0.01 vs. baseline.

Twenty-four patients with MetS participated in a GLP-1RA intervention study for 6 months (Figure 5(a)). Table S4 shows the main clinical features and metabolic parameters pre- and posttreatment. In comparison with pretreatment data, markers of lipid metabolism and obesity (BMI, FAT%, TG, TC, HDL-C, LDL-C, and LAP) and parameters associated with glucose metabolism and IR (HbA1c, FIns, and HOMA-IR) were significantly ameliorated 3 months posttreatment in patients with MetS (*p* < 0.01 or *p* < 0.05). Furthermore, 6 months posttreatment, FBG, 2 h-BG, and VAI also showed a significant decline (*p* < 0.01). A noticeable decline was observed in serum fetuin-B levels from 10.67 ± 4.87 mg/L pretreatment to 8.90 ± 3.45 mg/L 3 months posttreatment and 7.38 ± 2.74 mg/L 6 months posttreatment (Figure 5(b)). In addition, blood glucose levels at 120 min and the area under the curve for glucose (AUC_g_, 16.68 ± 2.79 vs. 19.46 ± 4.74 mmol/h/L, *p* < 0.05) during OGTTs were significantly lower than before GLP-1RA intervention (Figure 5(c)). In comparison with pretreatment data, *M* values during EHCs were significantly higher at both 3 and 6 months posttreatment (4.39 ± 1.30 and 4.66 ± 1.53 vs. 3.29 ± 0.82, *p* < 0.01 for all; Figure 5(d)). These data further confirmed that fetuin-B levels decreased *in vivo* with an improvement in IR.

## 4. Discussion

Fetuin-B, which is believed to be a hepatokine and/or adipokine, is mainly expressed in the liver and white adipose tissue, placenta, and heart, and it is evidently strongly associated with energy metabolism [6]. Several case-controlled and cross-sectional studies have reported that serum fetuin-B levels are markedly elevated in patients with nonalcoholic fatty liver disease, T2DM, gestational diabetes mellitus, and polycystic ovary syndrome [11–13]. However, only a few studies have explored the relationship between fetuin-B and MetS in humans, and the pathophysiological mechanism remains unclear. In this study, women were selected for eliminating the interference of gender differences. Serum fetuin-B levels were significantly elevated in women with MetS. In addition, fetuin-B was positively related to lipid- and glucose-related parameters and independently associated with MetS. As expected, in addition, fetuin-B overexpression aggravated oxidative stress *in vitro.* Therefore, our results suggest that fetuin-B impacts glucose and lipid metabolism and antioxidant stress, which is closely related to the occurrence and development of MetS. However, the cause of elevated fetuin-B levels in patients with MetS remains unknown. We believe that an increase in serum fetuin-B levels in patients with MetS can be attributed to elevated metabolic stress, involving, for example, hyperinsulinemia, dyslipidosis, and antioxidative disorder. These disorders stimulate fetuin-B release and secretion; nevertheless, further studies are warranted.

Meex et al. recently reported that fetuin-B silencing in obese mice improved glucose tolerance but did not affect body weight. In the case of humans, they found that serum fetuin-B was positively associated with fasting insulin and HOMA-IR, but no correlation was observed between serum fetuin-B and markers of obesity, inflammation, and blood fat [7]. Another study in women with gestational diabetes mellitus reported that serum fetuin-B had no association with obesity, hypertension, and dyslipidemia [13]. In addition, Qu et al. found that serum fetuin-B in patients with T2DM showed a significant positive association with TG, but no association was observed with other lipids or BMI [10]. However, herein, serum fetuin-B levels were noticeably elevated in overweight/obese women compared with lean women, and they were associated with BMI, LAP, and VAI; furthermore, TG and WHR were independently associated with serum fetuin-B. These data suggest that serum fetuin-B is associated with lipid metabolism and obesity. The precise reason for the discrepancy in our results and those reported by previous studies is unknown, but we believe that the discrepancy could be due to the higher BMI of patients with MetS included in this study. The association between fetuin-B and obesity thus remains ambiguous.

To further explore the association between fetuin-B and glucose and lipid metabolism, we performed bioinformatics analysis to identify related core genes and signaling pathways. Through the construction of a PPI network, we identified six fetuin-B-related proteins; they were enriched in pathways related to glucose and lipid metabolism, which formed the core of the PPI network. Among the identified proteins, alpha-2-HS-glycoprotein is reportedly associated with IR and diabetes [26, 27]; fibrinogen alpha chain with chronic inflammation, lipid metabolism, and diabetic complications [28]; and fibrinogen gamma chain and secreted phosphoprotein 24 with lipid metabolism and obesity [29]. Secreted phosphoprotein 24 is also involved in glucose and lipid metabolism [30]. Apolipoprotein A-IV and apolipoprotein B-100 are closely related to glucose and lipid metabolism, IR, and polycystic ovary syndrome [31–34]. Based on GO and KEGG pathway analysis, fetuin-B-related proteins were found to be involved in, for example, fat absorption and cholesterol metabolism. Therefore, consistent with the results of our population-based study, bioinformatics analysis revealed that fetuin-B is closely related to lipid metabolism and IR, ultimately leading to occurrence and development of MetS.

It has been well established that oxidative stress induced by lipid metabolism disorder is one of the important causes of IR [35]. However, whether fetuin-B promotes IR is related to oxidative stress remains unknown. In this experiment, we investigated the effect of fetuin-B on FFA-induced oxidative stress *in vitro*. The results demonstrated that fetuin-B overexpression aggravated oxidative stress by increasing ROS and MDA production and inhibiting SOD activity and GSH production. Meanwhile, Zhou et al. reported that hepatic knockdown of fetuin-B activated the AMP-activated protein kinase (AMPK) pathway to inhibit lipogenesis [21]. Fetuin-B silencing in obese mice improved glucose tolerance and IR [7]. This further supported our hypothesis that fetuin-B may aggravate metabolic disorder and IR in MetS individuals by promoting oxidative stress.

To evaluate whether blood glucose and insulin affect fetuin-B secretion, we performed OGTTs to observe changes in serum fetuin-B levels *in vivo.* A significant increase in serum fetuin-B levels was observed in healthy subjects, but patients with MetS showed no change. These results indicated that hyperglycemia and/or hyperinsulinemia promote fetuin-B secretion in healthy individuals.

EHC is the gold standard technique for evaluating IR in both humans and animals. During EHC, under the conditions of hyperinsulinemia and euglycemia, serum fetuin-B levels did not change in healthy subjects; however, a significant increase in serum fetuin-B levels was observed in patients with MetS. Therefore, we believe that hyperglycemia, not hyperinsulinemia, is the main factor affecting serum fetuin-B levels in healthy individuals. In patients with MetS, elevated insulin levels led to an increase in serum fetuin-B levels, while hyperglycemia inhibited fetuin-B release. Therefore, serum fetuin-B levels did not change during OGTTs under the conditions of hyperglycemia and hyperinsulinemia. However, the cause of this phenomenon is unknown. Meex et al. suggested that fetuin-B might regulate glucose metabolism by reducing glucose effectiveness or by another unknown insulin-independent mechanism [7]. Similar results were reported by another study [8]. In contrast, our human studies demonstrated that hyperglycemia may lead to an increase in fetuin-B levels in healthy women but inhibit fetuin-B release in patients with MetS. It remains unclear whether feedback regulation exists between fetuin-B and glucose. Moreover, as evident from our EHC results, *M* values showed significant differences between the groups. The difference between our results and those of previous studies could be attributed to long-term metabolic disorders and IR in women with MetS, but further studies need to be conducted to validate such findings.

Liraglutide is a GLP-1RA and is used for treatment of T2DM and obesity; it has beneficial effects on various metabolic parameters and is one of the preferred drugs for improving IR [36, 37]. In previous studies, we found that liraglutide promotes the secretion of some adipokines, such as adiponectin and visfatin *in vivo* [38, 39]. In this study, we found that GLP-1RA treatment for 6 months led to a significant decrease in serum fetuin-B levels, which was accompanied by ameliorated glucose metabolism and IR, as indicated by increased *M* values. Therefore, it is possible that chronic hyperinsulinemia related to IR results in an increase in fetuin-B levels. This indicates that the effect of liraglutide on fetuin-B levels is at least partially mediated by GLP-1RA-induced changes in insulin levels, which is secondary to the role of GLP-1RA in enhancing insulin sensitivity. These results further suggest that fetuin-B is associated with IR and MetS and also demonstrate a beneficial role of GLP-1RA in affecting fetuin-B secretion and release *in vivo*. Based on these data, it is apparent that fetuin-B levels tend to be lower in the state of insulin sensitivity and higher in the state of IR. Previous studies have found that GLP-1RA regulated the expression of lipid metabolism-related genes and delayed the cellular senescence by alleviating oxidative stress and inflammatory reaction [40]. A recent study has shown that liraglutide significantly reduced ROS production but increased SOD activity in high fat-treated HepG2 cells [41]. We found for the first time that the decrease of fetuin-B in MetS patients after liraglutide treatment may also be related to its antioxidation. However, more studies were needed to confirm our conjecture.

Our study has some limitations: (1) considering the cross-sectional design of this study, our results do not prove causal relationships; (2) we included a relatively small sample size, particularly in intervention experiments; therefore, our data may be affected by outliers; (3) this cohort included only Chinese women and did not include men. Therefore, our findings should be applied cautiously to other ethnic populations; and (4) the study failed to investigate the specific signal pathways by which fetuin-B affects hepatocytes. However, we included patients who were newly diagnosed with MetS and those without any lifestyle intervention and drug treatments, thereby avoiding the interference of disease course and other confounding factors. In addition, we performed various intervention experiments *in vivo* and *in vitro*, including EHC, to evaluate the association between fetuin-B and metabolism and IR. Thus, our data provide sufficient evidence to confirm that an association is present between fetuin-B and MetS in women.

## 5. Conclusions

We report that serum fetuin-B levels are elevated in women with MetS and that is related to glucose and lipid metabolism and IR. Using multiple interventions, including EHCs, OGTTs, and treatment with a GLP-1RA, we also found that serum fetuin-B was affected by blood glucose, insulin, and GLP-1RA *in vivo*. Additionally, fetuin-B overexpression aggravated oxidative stress *in vitro.* Therefore, we believe that fetuin-B can serve as a biomarker for screening MetS in women.

## Figures and Tables

**Table 1:** Main clinical features and serum fetuin-B levels in MetS and control subjects.

Variable	Controls (*n* = 185)	MetS (*n* = 192)	*p*
Age (years)^‡^	33.4 ± 13.1	42.2 ± 16.5	<0.001
BMI (kg/m^2^)	20.9 ± 2.7	26.3 ± 3.6	<0.001
FAT (%)	26.8 ± 5.3	37.3 ± 6.1	<0.001
WHR^‡^	0.80 ± 0.07	0.98 ± 0.34	<0.001
SBP (mmHg)	112.0 ± 13.3	129.4 ± 18.9	<0.001
DBP (mmHg)	73.3 ± 10.2	81.2 ± 12.1	<0.001
TC (mmol/L)	4.18 ± 1.01	4.79 ± 1.15	<0.001
TG (mmol/L)^†^	1.02 ± 0.58	2.27 ± 1.23	<0.001
HDL-C (mmol/L)^†^	1.35 ± 0.36	1.18 ± 0.34	<0.001
LDL-C (mmol/L)	2.37 ± 0.82	2.86 ± 0.88	<0.001
FFA (*μ*mol/L)	0.51 ± 0.23	0.63 ± 0.27	<0.001
HbA1c (%)^‡^	5.2 ± 0.3	6.2 ± 1.5	<0.001
FBG (mmol/L)^‡^	4.73 ± 0.52	6.55 ± 2.19	<0.001
2 h-BG (mmol/L)^†^	5.57 (4.85-6.44)	9.46 (7.55-12.02)	<0.001
FIns (mU/L)^†^	6.79 (5.70-8.28)	17.94 (11.46-27.51)	<0.001
2 h-Ins (mU/L)^†^	41.09 (26.13-60.29)	124.10 (67.76-221.90)	<0.001
HOMA-IR^†^	1.43 (1.16-1.78)	5.13 (3.42-7.51)	<0.001
LAP^†^	11.50 (5.36-20.71)	65.84 (48.29-89.02)	<0.001
VAI^†^	1.23 (0.85-1.74)	3.55 (2.58-4.81)	<0.001
Fetuin-B (mg/L)	6.01 ± 3.94	8.03 ± 3.75	<0.001
Fetuin-B (mg/L)^§^	6.07 ± 0.35	8.09 ± 0.33	<0.001

Values are given as mean ± SD or median (interquartile range). Abbreviations: BMI: body mass index; FAT%: the percentage of fat *in vivo*; WHR: waist-hip ratio; SBP: systolic blood pressure; DBP: diastolic blood pressure; TG: triglyceride; TC: total cholesterol; HDL-C: high-density lipoprotein cholesterol; LDL-C: low-density lipoprotein cholesterol; FFA: free fatty acid; FBG: fasting blood glucose; 2 h-BG: 2 h blood glucose after glucose overload; FIns: fasting plasma insulin; 2 h-Ins: 2 h plasma insulin after glucose overload; HbA1c: glycosylated hemoglobin; HOMA-IR: homeostasis model assessment of insulin resistance; LAP: lipid accumulation product; VAI: visceral adiposity index. ^†^Log transformed before analysis; ^‡^nonparametric tests; ^§^mean ± standard error by general linear model with adjustment of age and BMI.

## Data Availability

All the data used to support the findings of this study are available from the corresponding author upon request.

## Abbreviations

T2DM: Type 2 diabetes mellitus
MetS: Metabolic syndrome
OGTT: Oral glucose tolerance test
EHC: Euglycemic-hyperinsulinemic clamp
GLP-1RA: Glucagon-like peptide-1 receptor agonists
WHR: Waist-to-hip ratio
FAT%: Fat percentage *in vivo*
TG: Triglyceride
FBG: Fasting blood glucose
FIns: Fasting plasma insulin
HbA1c: Glycosylated hemoglobin
HOMA-IR: Homeostasis model assessment of insulin resistance
LAP: Lipid accumulation product
VAI: Visceral adiposity index
SOD: Superoxide dismutase
GSH: Glutathione
MDA: Malondialdehyde
ROS: Reactive oxygen species.

## Supplementary Materials

Supplementary Materials Table S1: correlation analysis of variables associated with circulating fetuin-B levels in study population. Table S2: association of serum fetuin-B levels with MetS in fully adjusted models. Table S3: row mean scores and Cochran–Armitage trend test of the impact of circulating fetuin-B levels on MetS individuals. Table S4: main clinical and metabolic features pre- and posttreatment with GLP-1RA in MetS women.
