# Supplementary material for: Serum Fetuin-B Levels Are Elevated in Women with Metabolic Syndrome and Associated with Increased Oxidative Stress
Source: Oxid Med Cell Longev. 2021 Oct 4;2021:6657658. doi: 10.1155/2021/6657658 (PMC8505080; doi:10.1155/2021/6657658)
Supplement: Supplementary Materials — Table S1: correlation analysis of variables associated with circulating fetuin-B levels in study population. Table S2: association of serum fetuin-B levels with MetS in fully adjusted models. Table S3: row mean scores and Cochran–Armitage trend test of the impact of circulating fetuin-B levels on MetS individuals. Table S4: main clinical and metabolic features pre- and posttreatment with GLP-1RA in MetS women. [file 6657658.f1.docx]

**Table S1** Correlation analysis of variables associated with circulating fetuin-B levels in study population.

| Variable | Simple | | Multiple | |
| --- | --- | --- | --- | --- |
|  | *r* | *p* | *B* | *P* |
| Age (years) ^‡^ | 0.094 | 0.070 | ------- | ------- |
| WHR^‡^ | 0.211 | < 0.001 | 1.731 | < 0.05 |
| BMI (kg/m^2^) | 0.154 | < 0.01 | ------- | ------- |
| FAT (%) | 0.209 | < 0.001 | ------- | ------- |
| SBP (mmHg) | 0.096 | 0.062 | ------- | ------- |
| DBP (mmHg) | 0.073 | 0.156 | ------- | ------- |
| TC (mmol/L ) | 0.070 | 0.180 | ------- | ------- |
| TG (mmol/L) ^†^ | 0.237 | < 0.001 | 0.674 | < 0.001 |
| HDL-C (mmol/L) ^†^ | -0.098 | 0.057 | ------- | ------- |
| LDL-C (mmol/L) | 0.070 | 0.177 | ------- | ------- |
| FFA (µmol/L) | -0.024 | 0.675 | ------- | ------- |
| HbA1c (%) ^‡^ | 0.146 | < 0.01 | ------- | ------- |
| FBG (mmol/L)^‡^ | 0.180 | < 0.001 | ------- | ------- |
| 2h-BG (mmol/L)^†^ | 0.136 | < 0.01 | ------- | ------- |
| FIns (mU/L)^†^ | 0.188 | < 0.001 | ------- | ------- |
| 2h-Ins (mU/L) ^†^ | 0.140 | < 0.01 | ------- | ------- |
| HOMA-IR † | 0.196 | < 0.001 | ------- | ------- |
| LAP † | 0.263 | < 0.001 | ------- | ------- |
| VAI ^†^ | 0.257 | < 0.001 | ------- | ------- |

Multiple linear stepwise regression analysis, values included for analysis were TG, WHR, BMI, HOMA-IR. ^†^, Log transformed before analysis; ^‡^, Spearman correlation tests;

**Table S2** Association of serum fetuin-B levels with MetS in fully adjusted models.

| **Model adjust** | **MetS** | | |
| --- | --- | --- | --- |
|  | OR | 95%CI | *P* |
| Age | 1.144 | 1.080 - 1.212 | < 0.001 |
| Age, BMI | 1.160 | 1.071 - 1.258 | < 0.001 |
| Age, BMI, FAT% | 1.136 | 1.045 - 1.236 | 0.003 |
| Age, BMI, FAT%, HbA1c | 1.133 | 1.034 - 1.242 | 0.008 |
| Age, BMI, FAT%, HbA1c, FIns | 1.142 | 1.023 - 1.274 | 0.018 |
| Age, BMI, FAT%, HbA1c, FIns, TC, LDL-C, FFA | 1.154 | 1.020 - 1.306 | 0.023 |

Results of binary logistic regression analysis are presented 95%CI, confidence interval; Abbreviations: BMI, body mass index; FAT%, fat percentage in vivo; TC, total cholesterol; LDL-C, low-density lipoprotein cholesterol; FFA, free fatty acid; TG, triglyceride; HbA1c, Glycosylated hemoglobin; FIns, fasting plasma insulin; OR, odds ratio.

**Table S3** Row mean scores and Cochran–Armitage trend test of the impact of circulating fetuin-B levels on MetS individuals.

|  | **MetS** | |
| --- | --- | --- |
|  | **χ²** | ***P*** |
| ROW Mean Scores Test | 18.4065 | < 0.001 |
| Cochran-Armitage Test | 17.7595 | < 0.001 |

**Table S4** Main clinical and metabolic features pre- and post-treatment with GLP-1RA in MetS women.

| Variable |  | Post-treatment | Post-treatment |
| --- | --- | --- | --- |
|  | Baseline | 3 months | 6 months |
| BMI (kg/m^2^) | 29.4 ± 2.9 | 27.1 ± 2.6^**^ | 26.7 ± 2.9^**^ |
| FAT (%) | 42.9 ± 8.3 | 37.2 ± 3.7^**^ | 37.1 ± 4.5^**^ |
| WHR | 0.90 ± 0.05 | 0.89 ± 0.05 | 0.89 ± 0.06 |
| SBP (mmHg) | 118.2 ± 9.9 | 113.5 ± 10.9 | 115.4 ± 12.1 |
| DBP (mmHg) | 76.0 ± 10.2 | 77.2 ± 9.8 | 77.4 ± 8.6 |
| TC (mmol/L) | 4.85 ± 0.91 | 4.19 ± 0.74^**^ | 4.20 ± 0.76^**^ |
| TG (mmol/L) | 2.31 ± 0.82 | 1.79 ± 0.63^*^ | 1.48 ± 0.69^**^ |
| HDL-C (mmol/L) | 1.06 ± 0.17 | 0.99 ± 0.18^*^ | 1.19 ± 0.71 |
| LDL-C (mmol/L) | 3.08 ± 0.80 | 2.63 ± 0.67^*^ | 2.53 ± 0.73^*^ |
| FFAs (µmol/L) | 0.54 ± 0.21 | 0.49 ± 0.17 | 0.47 ± 0.47 |
| FBG (mmol/L) | 5.63 ± 0.88 | 5.37 ± 0.43 | 5.26 ± 0.37^*^ |
| 2h-BG (mmol/L) | 9.56 ± 2.90 | 8.58 ± 2.45 | 7.39 ± 1.90^**^ |
| HbA1c (%) | 5.7 ± 0.5 | 5.4 ± 0.3^*^ | 5.3 ± 0.3^**^ |
| FIns (mU/L) | 29.05 (21.52 - 39.88) | 19.01 (12.92 - 30.55)^**^ | 16.75 (12.38 - 28.11)^**^ |
| 2h-Ins (mU/L) | 203.45 (157.93 - 348.98) | 219.25 (115.03 - 385.78) | 151.85 (80.63 - 250.10) |
| HOMA-IR | 7.31 (4.83 - 10.77) | 4.67 (3.05 - 7.12)^**^ | 4.18 (2.90 - 6.53)^**^ |
| LAP | 74.24 (50.99 - 107.27) | 50.98 (40.29 - 60.74)^**^ | 43.23 (22.76 - 63.05)^**^ |
| VAI | 3.77 (3.13 - 5.47) | 3.51 (2.39 - 4.68) | 2.51 (1.43 - 3.84)^**^ |
| M-values | 3.29 ± 0.82 | 4.39 ± 1.30^**^ | 4.66 ± 1.53^**^ |
| AUCg | 19.46 ± 4.74 | 17.52 ± 3.82 | 16.68 ± 2.79^*^ |
| Fetuin-B (mg/L) | 10.67 ± 4.87 | 8.90 ± 3.45 | 7.38 ± 2.74^*^ |

Values are given as mean ± SD or median (Inter quartile Range). AUCg, the area under the curve for glucose. **p* < 0.05, ***p* < 0.01 *vs.* Baseline.
